# Facilitating effects of the reductive soil disinfestation process combined with *Paenibacillus* sp. amendment on soil health and physiological properties of *Momordica charantia*

**DOI:** 10.3389/fpls.2022.1095656

**Published:** 2023-01-17

**Authors:** Liangliang Liu, Yi Xie, Xin Zhong, Quanquan Deng, Qin Shao, Zucong Cai, Xinqi Huang

**Affiliations:** ^1^Engineering Technology Research Center of Jiangxi Universities and Colleges for Selenium Agriculture, College of Life Science and Environmental Resources, Yichun University, Yichun, China; ^2^School of Geography, Nanjing Normal University, Nanjing, China; ^3^Jiangsu Center for Collaborative Innovation in Geographical Information Resource Development and Application, Nanjing Normal University, Nanjing, China; ^4^Jiangsu Engineering Research Center for Soil Utilization & Sustainable Agriculture, Nanjing Normal University, Nanjing, China

**Keywords:** soil health, microbial community, *paenibacillus*, probiotic consortia, fusarium wilt

## Abstract

Reductive soil disinfestation (RSD) is an anaerobic and facultative anaerobic microbial-mediated soil management process. The extent of improvement of diseased soil properties by RSD relative to comparable healthy soil is, however, not well characterized. Importantly, how to promote the colonization efficiency of these facultative anaerobic functional species to ensure soil and plant health remain unknown. Here, *Fusarium* wilt-diseased soil of *Momordica charantia* grown under a plastic-shed field (PS-CK) was used to conduct molasses-RSD (MO-RSD) along with *Paenibacillus* sp. (a model of facultative anaerobic species) (MO_PA_-RSD) treatment, and the soil from a nearby open-air paddy field was considered comparable healthy soil (OA-CK). Both RSD treatments significantly improved the properties of PS-CK soil, and the extent of improvement of soil pH, *Fusarium oxysporum* reduction efficiency (98.36%~99.56%), and microbial community and functional composition were higher than that achieved for OA-CK soil, which indicated that RSD-regulated most soil properties outperformed those of the comparable healthy soil. The disease incidence and ascorbic acid content of *M. charantia* in MO-RSD- and MO_PA_-RSD-treated soils were considerably decreased, while the weight and soluble protein contents were correspondingly increased, as compared to those of *M. charantia* in PS-CK soil. Specifically, the changes in these physiological properties of *M. charantia* in MO_PA_-RSD soil performed well than that in MO-RSD soil. The relative abundances of *Cohnella*, *Effusibacillus*, *Rummeliibacillus*, *Oxobacter*, *Thermicanus*, and *Penicillium* enriched in both RSD-treated soils were positively correlated with *Paenibacillus* and negatively correlated with *F. oxysporum* population and disease incidence (*P* < 0.05). Notably, the relative abundances of these potential probiotics were considerably higher in MO_PA_-RSD-treated soil than in MO-RSD alone-treated soil. These results show that the RSD process with inoculation of *Paenibacillus* sp. could promote the colonization of this species and simultaneously stimulate the proliferation of other probiotic consortia to further enhance soil health and plant disease resistance.

## Introduction

Driven by the shortage of land resources and economic benefits, the plastic shed cultivation system has become a popular soil utilization strategy in contemporary agriculture. Unfortunately, the management practices adopted in this cultivation system are usually characterized by overfertilization and intensive cultivation, thereby posing a serious threat to plant health through its adverse effects on soil properties (Fan et al., 2021; Shen et al., 2021). It is accepted that the soil microbial community that dominated by some beneficial functional microorganisms, such as rhizosphere growth-promoting bacteria (PGRP), can directly limit the invasion of pathogens into plant roots by competing for ecological resources, producing antagonistic substances, and inducing plant defense response (Raaijmakers et al., 2009; Mendes et al., 2011; Jin et al., 2022). Soil abiotic properties (e.g., pH and contents of salt, moisture, and nutrients) can indirectly influence plant health through their effects on microbial community composition (Janvier et al., 2007; Fierer, 2017; Liu et al., 2022a). Thus, effective management strategies for soil abiotic and biotic properties should be developed to maintain soil ecosystem health and ensure future food security.

Reductive soil disinfestation (RSD), also known as biological or anaerobic soil disinfestation, is defined as a process in which soil is incorporated with labile organic matter, irrigated to saturation, and covered with a plastic film to establish a strong anaerobic and reductive environment for improving soil physicochemical properties and microbial communities (Blok et al., 2000; Butler et al., 2012; Huang et al., 2016). Organic acids (acetic acid, propionic acid, butyric acid, and isovaleric acid) produced by functional microorganisms (such as *Clostridium*, *Coprococcus*, and *Ruminococcaceae*) during RSD treatment can significantly protect against multiple plant pathogens (Huang et al., 2015; Shrestha et al., 2016; Mao et al., 2022). To date, the RSD practice is widely considered an effective strategy for soil and plant health management and has been popularized in the Netherlands, China, USA, and Japan (Blok et al., 2000; Butler et al., 2012; Momma et al., 2013; Huang et al., 2016). Despite such promising results, the maximum improvement efficiency of soil properties by RSD remains unclear; this is because the current findings are obtained for diseased soils and lack the results for comparable healthy soils. Particularly, these studies on RSD aimed at alleviating the occurrence of plant diseases; however, how RSD affects other physiological properties of plants, especially predicators related to plant defense response, has not been well studied.

Because most fungi grow in an aerobic environment, the functional microorganisms during the RSD treatment process are dominated by anaerobic and facultative anaerobic bacteria such as most members of *Firmicutes* (Mowlick et al., 2013; Hewavitharana et al., 2019). The composition of these functional microorganisms in RSD-treated soils could be affected by the characteristics of organic matter, including decomposability, total carbon input, and carbon/nitrogen (C/N) ratio (Butler et al., 2012; Liu et al., 2016; Shrestha et al., 2021). For example, a higher abundance of *Firmicutes* was observed in RSD soil incorporated with readily decomposable organic matter (such as molasses and ethanol) than in RSD soil incorporated with plant residues, while soil without organic matter input (irrigated alone) showed no influence on *Firmicutes* composition (Liu et al., 2016; Hewavitharana et al., 2019). Additionally, although facultative anaerobic microorganisms continue to be the dominant taxa even after aerobic conditions are restored for RSD-treated soil, their abundance often shows a declining trend as compared to that during RSD treatment (Mowlick et al., 2013; Liu et al., 2018). Therefore, it is important to develop appropriate methods to promote the colonization efficiency of these native functional microorganisms, especially facultative anaerobic bacteria, in RSD-treated soils for maintaining soil and plant health. Our previous studies showed that biocontrol agents such as *Bacillus subtilis* SQR-N1 and *Trichoderma harzianum* SQR-T37 inoculated into the RSD-completed soil (aerobic conditions) could further enhance its resistance to damping-off and *Fusarium* wilt diseases of cucumber (Huang et al., 2016; Ali et al., 2022). However, we are unsure whether the functional microorganisms inoculated into the strongly anaerobic and reductive environment, which is the RSD treatment process, may improve their colonization efficiencies and plant disease resistance.

*Paenibacillus* sp. is an important PGRP and has been reported as a common facultative anaerobic species in the RSD practices that treated with different types of organic materials (Huang et al., 2018; Zhao et al., 2020; Liu et al., 2021). Specifically, our previous study has demonstrated that the relative abundance of *Paenibacillus* sp. in the RSD incorporated with liquid-readily decomposable compounds (such as molasses) was significantly higher than that amended with solid plant residues (such as sawdust) (Yan et al., 2022). In the present study, therefore, the diseased soil of *Momordica charantia* severely infected with *Fusarium oxysporum* f. sp. *momordicae* cultivated under the plastic shed field (PS-CK) was used to conduct molasses-RSD (MO-RSD) along with *Paenibacillus* sp. inoculation (MO_PA_-RSD); soil from the nearby open-air paddy field (OA-CK) was considered a comparable healthy or original soil. The study aimed to address the above-mentioned knowledge gaps: (1) to what extent does RSD improve the properties of PS-CK soil relative to those of OA-CK soil, (2) whether the addition of facultative anaerobic functional microorganisms during RSD treatment is more conducive to manage plant physiological properties, and (3) what are the underlying drivers that could be revealed by deciphering changes in soil microbial communities after treatment?

## Materials and methods

### Field description

The plastic shed field was located in Nanmiao Town (28^°^9′ N, 114^°^42′ E), Jiangxi Province, China. Limited rotation of *M. charantia* and edible amaranth is the primary cultivation pattern in this field, while the incidence of *Fusarium* wilt disease of *M. charantia* has exceeded 30% in recent seasons. The classification of this soil is a Feeralic Cambisol (IUSS Working Group WRB, 2015), with the following initial properties: total organic carbon (TOC) 26.02 g kg^–1^; total nitrogen (TN) 3.52 g kg^–1^; and pH 4.11 (Liu et al., 2022b).

### Experimental design

Four treatments with a randomized complete block design were conducted in this study: OA-CK, untreated flooded paddy soil in the open-air cultivation system; PS-CK, untreated soil (converted from OA-CK 4 years ago) in the plastic shed field with moisture content maintained at 15~20%; MO-RSD, PS-CK soil amended with 7.5 t ha^-1^ molasses (TOC 347.8 g kg^–1^, TN 16.8 g kg^–1^, and C/N ratio 20.7), irrigated to saturation, and covered with a plastic film (transparent, 0.08 mm thickness); and MO_PA_-RSD, MO-RSD treatment process combined with inoculation of *Paenibacillus* sp. agent (2.5 × 10^10^ copies g^-1^ m^-2^) provided by Beihai Ye Sheng Wang Biological Texhnology Co., LTD, Guangxi Province, China (accession number: OP602366). The molasses and *Paenibacillus* sp. agent were diluted to 5% before use. Each treatment contained three replicates, and the area of each replicate covered 60 m^2^. The treatment was followed for 18 days at an average greenhouse temperature of 28~40 °C (randomly measured by a thermometer). The additive amount of molasses and incubation period and temperature during RSD treatment were according to our previous study (Yan et al., 2022), which has demonstrated that RSD treatment under the above conditions can significantly improve soil quality by improving soil microbial community and function. The plastic films in the RSD treatments were then removed, and the soils were then drained. Soil samples (0~15 cm depth) in each replicate were collected from 6 soil cores and stored at 4°C for analyzing physicochemical properties and at -20 °C for DNA extraction. Seedlings of *M. charantia* with three leaves were planted in PS-CK-, MO-RSD-, and MO_PA_-RSD-treated soils at intervals of 50 cm. After 90 days, the disease incidence in each soil was recorded. The fruits, ranked from largest to smallest in weight (**Figure S1**), were randomly collected from each replicate to detect the physiological properties.

### Determination of soil physicochemical and plant physiological properties

Soil pH and electrical conductivity (EC) were measured using the S220K and S230K electrodes (Mettler-Toledo International Inc., Shanghai, China) with a soil: water (w/v) ratio of 1:2.5 and 1:5, respectively. Soil ${\text{NO}}_{3}^{-}$ -N and ${\text{NH}}_{4}^{+}$ -N were extracted using KCl solution (2 mol L^-1^) with a ratio of soil: solution of 1:5 and detected by a continuous flow analyzer (San++; Skalar, Breda, the Netherlands).

The disease incidence, fruit weight, ascorbic acid content, soluble protein content, and texture properties (hardness and fracturability) of fruits were used to indicate the physiological properties of the plant. Ascorbic acid content was measured with 2,6-dichlorophenol indophenol using the colorimetric method with the measurement of absorbance at 520 nm (Niroula et al., 2021). Soluble protein content was estimated using the Coomassie Brilliant Blue G-250 dye as described by Bradford (1976), and bovine serum albumin was used to generate the protein standard. Hardness and fracturability of the fruit were determined using a 2-mm-diameter stainless steel cylinder probe at the speed of 1 mm s^-1^ on a texture analyzer (TA.XTC-18, BosinTech Co., Ltd., Shanghai, China). All measurements were conducted in triplicate to avoid the randomness of the data.

### DNA isolation and microbial quantification

Soil microbial DNA was isolated from each replicate (0.5 g soil) using the FastDNA Spin Kit (MP Biomedicals, Santa Ana, CA, USA), and the quality of the isolated DNA was assessed by a DS-11 spectrophotometer (Denovix Inc., Wilmington, DE, USA). Bacteria, fungi, and *F. oxysporum* were quantified using the QuanStudio 3 Real-Time PCR system (Applied Biosystems, USA). All PCR reaction mixtures contained 1 µL of paired primers (**Table S1**), 2 µL of template DNA, 10 µL of SYBR Green premix Taq (2×), and 6 µL of sterile distilled water. Amplification protocols, specificity, and standard curves of each gene were established according to a previous study (Yan et al., 2022).

### MiSeq sequencing and data processing

The V4–V5 region of bacterial 16S rDNA and the internal transcribed spacer (ITS) region of fungi in all pre-planting soil samples (4 treatments × 3 replicates) were amplified using the paired primers 515F/907R and ITS1F/ITS2R, respectively (**Table S1**). All PCR reactions were performed using 15 µL of Phusion High-Fidelity PCR Master Mix (New England Biolabs), 2 µM of paired primers, and 10 ng template DNA. The equimolar concentrations of amplicons were then sequenced by Beijing Novogene Bioinformatics Technology Co. Ltd. (Beijing, China) on an Illumina NovaSeq platform.

Quantitative insights into microbial ecology 2 (QIIME2) (Bolyen et al., 2019) software was used to process the raw sequencing data along with the customized program scripts (https://docs.qiime2.org/2019.1/). Briefly, the files of paired-end FASTQ sequences were imported into the format that could be recognized by the QIIME2 system using the qiime tools import program. Demultiplexed sequences from each sample were quality-filtered, trimmed, de-noised, and merged. The chimeric, contaminating mitochondrial, and chloroplast sequences were then identified and removed using the QIIME2 dada2 plugin to obtain the feature table of amplicon sequence variant (ASV) (Callahan et al., 2016). The high-quality bacterial and fungal ASV sequences were rarefied to 36,429 and 40,584 sequences and then annotated according to pre-trained databases of SILVA (Quast et al., 2013) and UNITE (Kõljalg et al., 2013) at 99% similarity by using the QIIME2 feature-classifier plugin, respectively.

### Bioinformatics and statistical analyses

Microbial α diversity was calculated using the core-diversity plugin in QIIME2. Microbial β diversity based on Bray–Curtis distance matrices at the genus level was analyzed by principal coordinate analysis (PCoA) using the Wekemo Bioincloud (https://www.bioincloud.tech). The effect of different treatments on microbial β diversity was calculated by permutational multivariate analysis of variance (PERMANOVA) using the “adonis” function in R “vegan” package (Oksanen et al., 2022). Moreover, because the microbial communities between OA-CK and PS-CK soils or MO-RSD- and MO_PA_-RSD-treated soils have certain similarities, the interactions of bacterial or fungal taxa between CK- (PS-CK and OA-CK) and RSD- (MO-RSD and MO_PA_-RSD) treated soils were determined using the co-occurrence network analysis. In brief, bacterial and fungal ASV sequences with abundances lower than 0.15% and 0.1% were filtered, and Pearson’s correlations with a cutoff at |*r*| > 0.95 and *P*-value < 0.01 (corrected by false discovery rate) among the obtained taxa were calculated using R “psych” package (Steinhauser et al., 2007). Gephi (Bastian et al., 2009) software (version 0.9.2) was then used to visualize the network composition and determine the topology parameters. To gain insights into the effect of RSD on soil microbial functions, PICRUSt2 and FUNGuild were used to infer Kyoto Encyclopedia of Genes and Genomes (KEGG) and ecological guild functional profiles based on the bacterial 16S rDNA and fungal ITS ASV sequences, respectively (Langille et al., 2013; Nguyen et al., 2016). The relationships between soil dominant genera and plant physiological properties were visualized by the partial mantel test and network interaction analysis according to Liu et al. (2022a).

Significant differences (*P* < 0.05) between plant physiological properties, soil physicochemical properties, microbial abundance (transformed by 1og_10_), and dominant genera in different treatments were determined by one-way analysis of variance (ANOVA) and LSD test using SPSS 22.0 (SPSS Inc., Chicago, IL).

## Results

### Soil physicochemical properties

The pH of PS-CK soil was significantly decreased (*P* < 0.05) by 0.95 as compared to that of OA-CK soil, while the opposite result was observed following the treatment of PS-CK soil with MO-RSD and MO_PA_-RSD, wherein the pH of these soils remarkably increased (*P* < 0.05) by 1.16 and 1.23, respectively, and was significantly higher than that of OA-CK soil (**Table 1**). Compared to OA-CK soil, PS-CK soil showed a significant increase (*P* < 0.05) in EC and ${\text{NO}}_{3}^{-}$ -N by 0.38 mS cm^-1^ and 134 mg kg^-1^, respectively (**Table 1**), while these properties were considerably (*P* < 0.05) decreased in RSD-treated soils (**Table 1**). Specifically, soil ${\text{NO}}_{3}^{-}$ -N were at a similar level (*P* > 0.05) between OA-CK and RSD-treated soils. The ${\text{NH}}_{4}^{+}$ -N content in the RSD-treated soils was significantly higher (*P* < 0.05) than that in CK soils (**Table 1**).

**Table 1:** Soil physicochemical properties in different treatments.

Treatment ^a^	pH ^b^	EC (mS cm^-1^) ^c^	${\text{NO}}_{3}^{–}$ N (mg kg^-1^)	${\text{NH}}_{4}^{+}$ -N (mg kg^-1^)
PS-CK	4.11 ± 0.11c	0.41 ± 0.33a	135.0 ± 2.25a	55.55 ± 16.41b
OA-CK	5.06 ± 0.09b	0.03 ± 0.00d	1.00 ± 0.36b	3.15 ± 0.25c
MO-RSD	5.27 ± 0.07a	0.11 ± 0.01c	0.41 ± 0.11b	88.15 ± 5.27a
MO_PA_-RSD	5.34 ± 0.08a	0.15 ± 0.02b	0.48 ± 0.14b	101.75 ± 11.79a

a PS-CK, untreated soil under the plastic shed field system; OA-CK, untreated flooded paddy soil under the open-air cultivation system; MO-RSD, PS-CK soil amended with 7.5 t ha^-1^ molasses, irrigated to saturation, and covered with a plastic film; MO_PA_-RSD, MO-RSD treatment process combined with *Paenibacillus* sp. agent inoculation.

b Values (means ± SD, n = 3) followed by different letters in each column represent significant differences at *P* < 0.05 according to LSD test.

c EC, electrical conductivity.

### Populations of soil bacteria, fungi, and *F. oxysporum*

**Figure 1:**
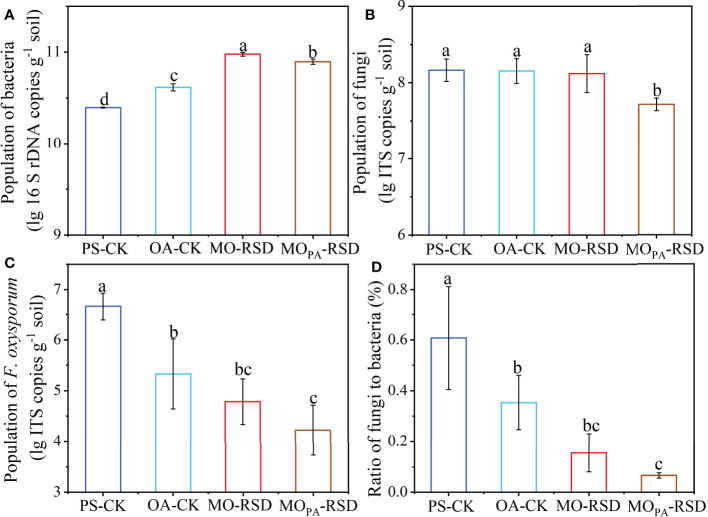
The populations of bacteria **(A)**, fungi **(B)**, and *F. oxysporum* **(C)** as well as the ratio of fungi to bacteria **(D)**. Error bars represent standard deviations (SDs). Different letters between various treatments represent significant differences at *P* < 0.05 according to LSD test. The treatment abbreviations are defined in **Table 1**.

The population of bacteria in PS-CK soil (2.49 × 10^10^ copies g^-1^) was significantly decreased (*P* < 0.05) by 0.39-fold as compared to that in OA-CK soil (4.15 × 10^10^ copies g^-1^), while the bacterial abundance was considerably increased (*P* < 0.05) by 2.81- or 2.16-fold and by 1.28- or 0.90-fold in MO-RSD-treated soil (9.50 × 10^10^ copies g^-1^) and MO_PA_-RSD-treated soil (7.89 × 10^10^ copies g^-1^) when compared with those in PS-CK and OA-CK soils, respectively (**Figure 1A**). No significant difference (*P* > 0.05) was observed in the population of fungi between PS-CK, OA-CK, and MO-RSD, while the fungal abundance was remarkably decreased (*P* < 0.05) by 65.53% after PS-CK soil (1.51 × 10^8^ copies g^-1^) was treated with MO_PA_-RSD (5.21 × 10^7^ copies g^-1^) (**Figure 1B**). The population of *F. oxysporum* in PS-CK soil (5.13 × 10^6^ copies g^-1^) was significantly increased (*P* < 0.05) by 90.43% as compared to that in OA-CK soil (4.91 × 10^5^ copies g^-1^), whereas the population of this pathogen was remarkably decreased (*P* < 0.05) by 98.36% and 99.56% after PS-CK soil was treated with MO-RSD (8.43 × 10^4^ copies g^-1^) and MO_PA_-RSD (2.29 × 10^4^ copies g^-1^), respectively (**Figure 1C**). The fungi/bacteria ratio in OA-CK and RSD-treated soils was significantly lower than that in PS-CK soil, while the lowest fungi/bacteria ratio was detected in MO_PA_-RSD soil (**Figure 1D**). Notably, these parameters of MO_PA_-RSD-treated soil were lower than those of MO-RSD-treated soil.

### Soil microbial α and β diversities

**Figure 2:**
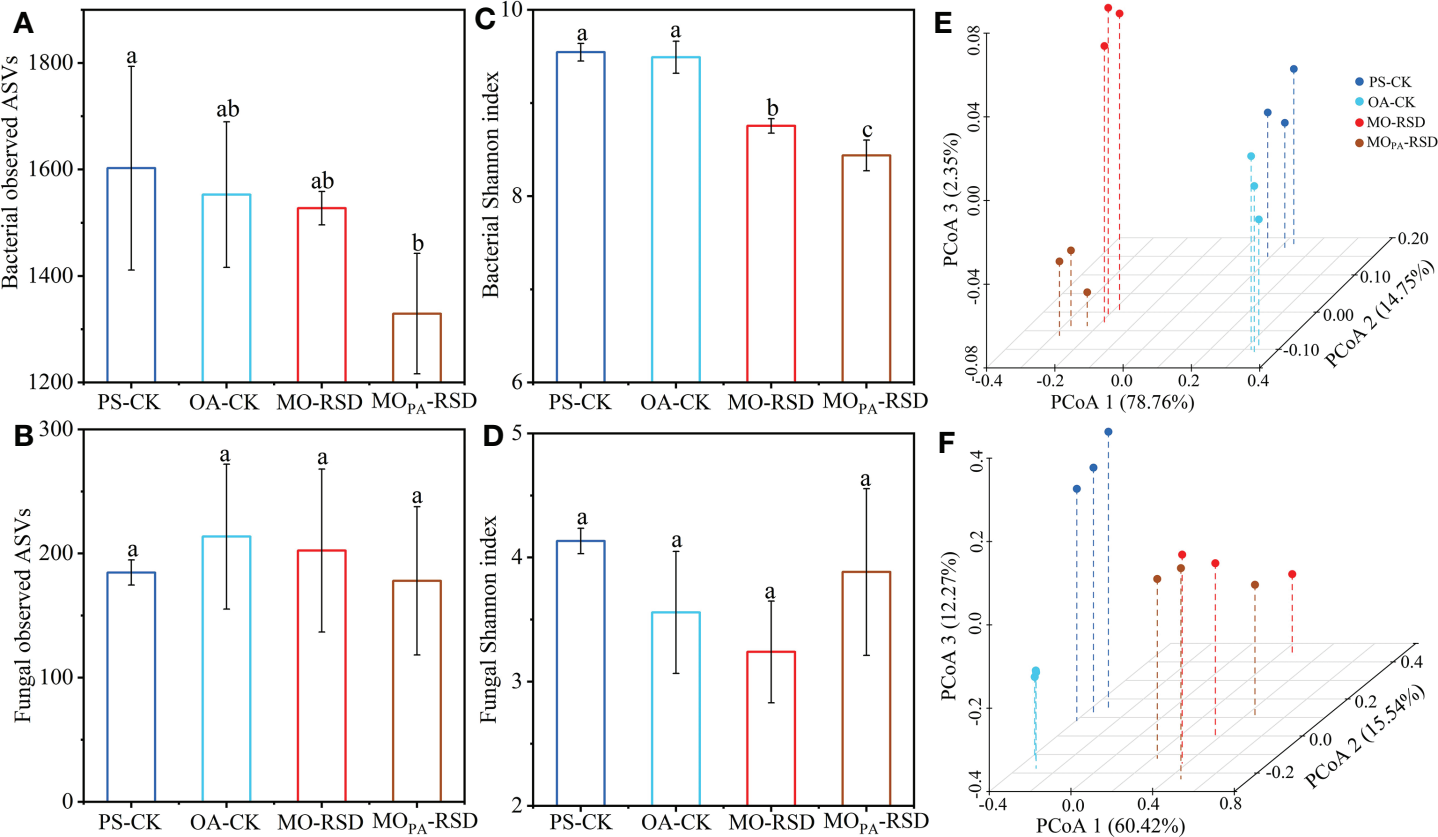
The microbial α **(A-D)** and β **(E, F)** diversities in the different treatments. Principal coordinates analyses (PCoAs) of the bacterial **(E)** and fungal **(F)** community structures were determined using the Bray-Curtis distance indices at the genus level. Error bars represent SDs. Different letters between various treatments represent significant differences at *P* < 0.05 according to LSD test. The treatment abbreviations are defined in **Table 1**.

Bacterial richness (observed species) in MO_PA_-RSD-treated soil was significantly decreased (*P* < 0.05) as compared to that in PS-CK soil, whereas bacterial richness in the remaining soils and fungal richness in all soils were not significantly different (*P* > 0.05) (**Figures 2A, B**). Bacterial Shannon index between PS-CK and OA-CK soils and fungal Shannon index in all soils showed no significant difference (*P* > 0.05), whereas RSD treatments remarkably decreased the bacterial Shannon index as compared to that in PS-CK soil (**Figures 2C, D**).

PCoA plots showed that the microbial community was significantly different (*P* < 0.01 according to PERMANOVA) between RSD-treated soils and CK soils (**Figures 2E, F**). Specifically, the dissimilarities in bacterial and fungal community structures between PS-CK and RSD-treated soils (determined by PC 1 scores: 78.76% and 60.42%, respectively) were larger than those between PS-CK and OA-CK soils (determined by PC 2 and PC 3 scores: 14.75% and 12.27%, respectively). The bacterial and fungal communities in MO_PA_-RSD-treated soil were slightly changed as compared to those in MO-RSD-treated soil, as determined by PC 3 (2.35%) and PC 2 (15.54%) scores, respectively.

### Co-occurrence network of soil microbial community

**Figure 3:**
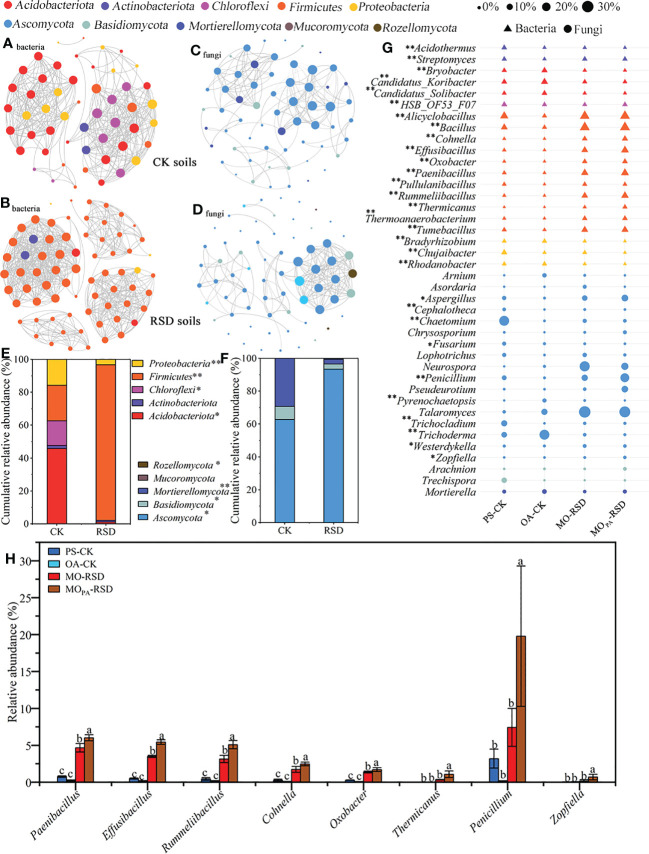
Dissimilarities in dominant microbial compositions among the different soils. Co-occurrence networks of bacterial **(A, B)** and fungal **(C, D)** dominant taxa were calculated using the Pearson correlations with a cutoff at |*r*| > 0.95 and *P*-value < 0.01 (FDR corrected), and the cumulative relative abundances of these nodes at the phylum level were showed in plots **(E, F)**. Relative abundances of the top 20 dominant bacterial and fungal genera **(G)** and that significantly enriched in RSD-treated soils **(H)** were listed. “*” (*P* < 0.05) and “**” (*P* < 0.01) indicate significant differences using the LSD test. Error bars in plot **(H)** represent SDs, and the different letters represent significant differences at *P* < 0.05 according to LSD test. CK or RSD soils indicate the combinations of PS-CK and OA-CK or MO-RSD and MO_PA_-RSD soils that are defined in **Table 1**.

Co-occurrence network analysis showed that the microbial interactions in RSD-treated soils (MO-RSD and MO_PA_-RSD) were significantly different as compared to those in CK soils (PS-CK and OA-CK) (**Figures 3A-D**). Specifically, the interactions of bacterial and fungal species in RSD-treated soil primarily included the members of *Firmicutes* and *Ascomycota* that accounted for >90% of the obtained sequences and more than that in the CK soils, respectively (**Figures 3E, F**). In addition, compared to CK soils, the RSD-treated soils showed higher topological characteristics of the bacterial network, such as number of nodes and edges, average connectivity, modularity, average path length, and average clustering coefficient; however, the fungal network in both RSD-treated soils showed an opposite trend for number of edges, average connectivity, modularity, and average path length (**Table S2**).

### Soil microbial community composition

The dominant bacterial phyla, accounting for 87.48~96.63% of all sequences, across all treatments were *Firmicutes*, *Acidobacteriota*, *Proteobacteria*, *Chloroflexi*, *Actinobacteriota*, *Gemmatimonadota*, *Desulfobacterota*, *RCP2_54*, *Bacteroidota*, and *Myxococcota* (**Figure S2A**). The relative abundances of all the phyla, except for *RCP2_54*, were significantly different (*P* < 0.05) between various treatments. Specifically, the relative abundance of *Firmicutes* was remarkably increased (*P* < 0.05) in RSD-treated soils as compared to that in PS- and OA-CK soils, and its relative abundance in MO_PA_-RSD-treated soil was considerably higher (*P* < 0.05) than that in MO-RSD-treated soil (**Figure S2A**). Furthermore, the relative abundances (accounting for 99.44~100%) of all dominant fungal phyla showed no significant difference between PS-CK and OA-CK soils (*P* > 0.05), whereas the relative abundance of *Ascomycota* was significantly (*P* < 0.05) increased after PS-CK soil was treated with MO-RSD and MO_PA_-RSD (**Figure S2B**).

The compositions of bacterial and fungal genera were clustered into CK- (OA-CK and PS-CK) and RSD- (MO-RSD and MO_PA_-RSD) groups, indicating most of these genera had a similar level of relative abundance between the groups (**Figure S3A, B**). However, the relative abundances of the dominant bacterial genera *Candidatus_Solibacter*, *Candidatus_Koribacter*, *Bryobacter*, and *Rhodanobacter* as well as the dominant fungal genera *Arnium*, *Trichoderma*, and *Pyrenochaetopsis* in PS-CK were significantly lower (*P* < 0.05) than those in OA-CK, whereas the genera *Chujaibacter*, *HSB_OF53_F07*, *Cephalotheca*, *Trichocladium*, *Chaetomium*, and *Fusarium* showed an opposite trend (**Figure 3G**). The relative abundances of the dominant bacterial genera *Alicyclobacillus*, *Bacillus*, *Cohnella*, *Effusibacillus*, *Oxobacter*, *Paenibacillus*, *Pullulanibacillus*, *Rummeliibacillus*, *Thermicanus*, *Thermoanaerobacterium*, and *Tumebacillus* were considerably increased (*P* < 0.05) in both RSD-treated soils as compared to those in both CK soils (**Figure 3G**). The relative abundances of the dominant fungal genera *Westerdykella* in MO-RSD-treated soil and *Zopfiella*, *Penicillium*, and *Aspergillus* in MO_PA_-RSD-treated soil were significantly higher (*P* < 0.05) than those in both CK soils. Interestingly, the relative abundances of *Paenibacillus*, *Cohnella*, *Effusibacillus*, *Rummeliibacillus*, *Oxobacter*, *Thermicanus*, *Zopfiella*, and *Penicillium* in MO_PA_-RSD-treated soil were remarkably higher (*P* < 0.05) than those in MO-RSD-treated soil (**Figure 3H**).

### Soil microbial functional composition

**Figure 4:**
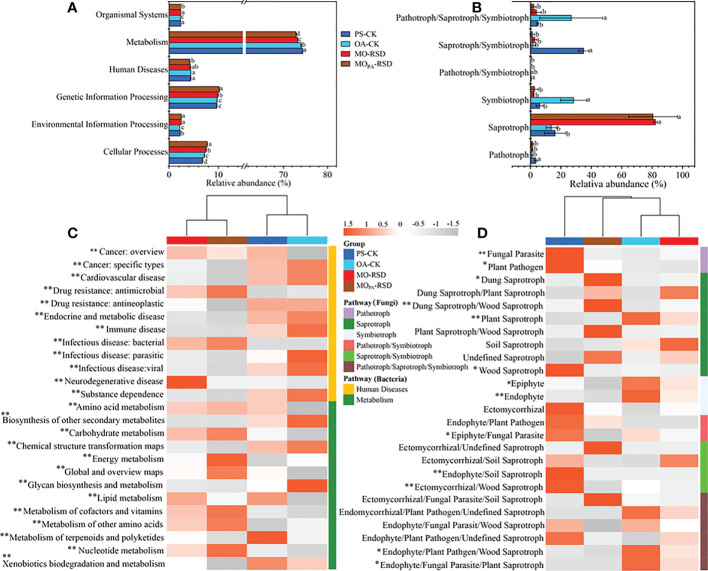
Dissimilarities in bacterial KEGG orthologue and fungal ecology guild functional profiles among the different treatments. The bacterial (**A**, level 2; **C**, level 3) and fungal (**B**, level 1; **D**, level 2) functional profiles were predicted by PICRUSt2 and FUNGuild, respectively. The dominant pathway of human diseases and metabolism profiles **(C)** in bacteria were listed. Error bars in plots **(A, B)** represent SDs, and the different letters represent significant differences at *P* < 0.05 according to LSD test. The key from gray to red indicate the least abundant to most abundant in each row for a given functional profile **(C, D)**, and “*” (*P* < 0.05) and “**” (*P* < 0.01) indicate significant differences using LSD test. The treatment abbreviations are defined in **Table 1**.

Compared to OA-CK soil, PS-CK soil showed a significant decrease (*P* < 0.05) in the relative abundances of bacterial functions such as cellular processes and fungal functions such as symbiotroph and pathotroph/saproytroph/symbiotroph; in contrast, the relative abundances of environmental information processing, metabolism, and organismal system in the bacterial function profile and pathotroph and saprotroph in the fungal function profile of PS-CK soil showed a significant upward trend (*P* < 0.05) (**Figures 4A, B**). Additionally, the relative abundances of bacterial functions such as cellular processes, environmental information processing, and genetic information processing and fungal functions such as saprotroph were remarkably increased in RSD-treated soils as compared to those in PS-CK soil, while the relative abundances of human diseases, metabolism, and organismal systems in the bacterial function profile as well as pathotrophs in the fungal function profile showed an opposite trend (**Figures 4A, B**). Specifically, the relative abundances of cellular processes and genetic information processing in MO_PA_-RSD-treated soil were significantly higher than those in MO-RSD-treated soil, while the relative abundances of metabolism and organismal systems were significantly higher in MO-RSD-treated soil (**Figures 4A, B**).

In the bacterial functional pathway of human diseases (**Figure 4C**), we found that the relative abundances of drug resistance: antimicrobial and bacterial infectious disease were significantly enriched (*P* < 0.05) in RSD-treated soils, whereas the relative abundances of other human diseases, i.e., cancer: specific types, drug resistance: antineoplastic, immune disease, substance dependence, etc., were remarkably enriched (*P* < 0.05) in CK soils. Regarding metabolism (**Figure 4C**), we found that the relative abundances of amino acid metabolism, carbohydrate metabolism, energy metabolism, global and overview maps, metabolism of cofactors and vitamins, metabolism of other amino acids, and nucleotide metabolism were significantly increased (*P* < 0.05) in RSD-treated soils, whereas the relative abundances of biosynthesis of other secondary metabolites and xenobiotic biodegradation and metabolism were higher (*P* < 0.05) in CK soils. In the fungal functional pathway of pathotroph (**Figure 4D**), we found that the relative abundances of fungal parasites and plant pathogens in PS-CK soil were significantly higher (*P* < 0.05) than those in other soils. For saprotrophs, the relative abundances of dung, dung/wood, and plant/wood saprotrophs in MO_PA_-RSD-treated soil and the relative abundances of dung/plant, plant, and soil saprotrophs in MO-RSD-treated soil were higher than those in PS-CK soil. For symbiotrophs, we observed that the relative abundances of epiphytes and endophytes in OA-CK soil were considerably higher (*P* < 0.05) than those in other soils.

### Plant physiological properties

Compared to PS-CK soil, both MO-RSD- and MO_PA_-RSD-treated soils showed a significant decrease (*P* < 0.05) in the disease incidence of the *M. charantia* plant by 75.21% and 90.09%, respectively (**Table 2**). The ascorbic acid content, hardness, and fracturability of *M. charantia* fruits grown in MO-RSD and MO_PA_-RSD soils were significantly decreased (*P* < 0.05) by 61.18% and 74.34%, 14.73% and 37.98%, and 20.23% and 41.17%, respectively (**Table 2**). Conversely, the soluble protein content and weight of fruits grown in MO-RSD- and MO_PA_-RSD-treated soils were significantly increased (*P* < 0.05) by 0.89- and 1.08-fold and by 0.64- and 1.04-fold, respectively, when compared with those of fruits grown in PS-CK soil (**Table 2**). Notably, the weight and soluble protein content (*P* < 0.05) of fruits grown in MO_PA_-RSD-treated soil were higher than those of fruits grown in MO-RSD soil, while the disease incidence, ascorbic acid, hardness (*P* < 0.05), and fracturability (*P* < 0.05) of fruits grown in MO_PA_-RSD-treated soil showed an opposite trend.

**Table 2:** Plant physiological properties in different soils.

Physiological properties	PS-CK ^a^	MO-RSD	MO_PA_-RSD
Disease incidence (%)	44.83 ± 2.75a	11.11 ± 3.84b	4.44 ± 3.84b
Ascorbic acid (mg kg^-1^)	183.81 ± 32.26a	71.34 ± 15.21b	47.16 ± 1.81b
Soluble protein (mg g^-1^)	1.69 ± 0.15c	3.19 ± 0.09b	3.51 ± 0.17a
Weight (g fruit^-1^)	66.87 ± 15.78b	109.71 ± 13.16a	136.10 ± 25.73a
Hardness (N)	6.61 ± 0.42a	5.63 ± 0.43b	4.09 ± 0.37c
Fracturability (N)	1.70 ± 0.16a	1.36 ± 0.06b	1.00 ± 0.02c

a Values (means ± SD, n = 3) followed by different letters in each row represent significant differences at *P* < 0.05 according to LSD test. The treatment abbreviations are defined in **Table 1**.

### Soil microbial composition associated with plant physiological properties

**Figure 5:**
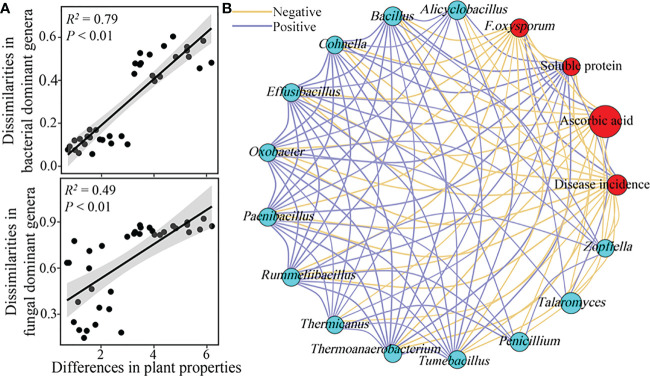
Soil microbial composition associated with plant physiological properties. Relationships between the dissimilarities in relative abundances of bacterial and fungal **(A)** dominant genera and the differences in plant properties were calculated using partial mantel test. The differences in plant properties were normalized according to the z-score method. Network interactions among the relative abundances in dominant genera that significantly enriched in RSD-treated soils, *F. oxysporum population*, disease incidence, and contents of soluble protein and ascorbic acid of plants were listed in plot **(B)**.

The dissimilarities in the dominant bacterial and fungal genera composition were significantly and positively correlated with the differences in plant physiological properties, and the effects of the dominant bacterial genera composition on plant physiological properties were larger than those of the dominant fungal genera composition (**Figure 5A**). Specifically, the population of *F. oxysporum*, plant disease incidence, and ascorbic acid content showed a significant and positive interaction with each other, while the opposite trend was observed between these properties and soluble protein content (**Figure 5B**). Furthermore, the relative abundances of the dominant genera that increased in RSD-treated soils, such as *Alicyclobacillus*, *Bacillus*, *Cohnella*, *Effusibacillus*, *Oxobacter*, *Paenibacillus*, *Rummeliibacillus*, *Thermicanus*, *Thermoanaerobacterium*, *Tumebacillus*, *Penicillium*, *Talaromyces*, and *Zopfiella*, were significantly and negatively (*P* < 0.05) correlated with the populations of *F. oxysporum*, disease incidence, and ascorbic acid content (**Figure 5B**).

## Discussion

### RSD-regulated most soil properties outperformed those of the comparable healthy soil

Soil acidification and secondary salinization are the two major degradation characteristics in the plastic-shed cultivation system due to long-term overfertilization (Fan et al., 2021; Zhang et al., 2022), similar to pH and EC of PS-CK soil in the present study. The pH and ${\text{NH}}_{4}^{+}$ -N content of RSD-treated soils, especially those of MO_PA_-RSD-treated soil, were significantly higher than those of CK soils, while the EC and ${\text{NO}}_{3}^{-}$ -N content of the former group were remarkably decreased to a level comparable to those of OA-CK soil; this finding indicates that the restored effects of RSD on soil acidification outperformed that in OA-CK soil. This was mainly due to the reduction and denitrification environment created during RSD treatment, which may be stronger than that in the flooded paddy field, thus leading to rapid consumption of H^+^ in the soil (Zhu et al., 2011; Di Gioia et al., 2017).

Microbial abundance, diversity, community, and functional compositions have long been considered the important predictors of soil health (Chaparro et al., 2012; Wang et al., 2019). Although the increase in bacteria and decrease in plant pathogens after RSD treatment are a common phenomenon, these properties showed a better performance than those in OA-CK soil, which was supported by molasses used in RSD treatment that can stimulate the proliferation of beneficial bacteria and produce a high amount of organic acids (Butler et al., 2012; Momma et al., 2013). The microbial communities in healthy soils often have highly connected networks that can directly resist the invasion of plant roots by pathogens (Wang et al., 2019; Wei et al., 2019). Herein, we observed that the bacterial network in RSD-treated soils was more complex and highly connected than that in CK soils, indicating the changes in the bacterial community during RSD treatment play a predominant role in maintaining good soil health. These results were further supported by significant enrichment of the beneficial members of *Firmicutes* in the RSD-treated network and the larger dissimilarities in bacterial communities between RSD-treated soils and PS-CK soils than that between OA-CK and PS-CK soils. Notably, previous studies have revealed that the dominant microbes belonging to *Firmicutes* significantly increased during the RSD treatment can result in a decrease in soil bacterial α diversity (Meng et al., 2019; Huang et al., 2018), which may also be the main reason for the change of bacterial diversity in this study.

The downward trend of the total metabolism activity in RSD-treated soil was contrary to that observed in previous studies (Zhao et al., 2020; Li et al., 2021); this was mainly because that the metabolism of all soil microorganisms was detected in these previous studies by using Biolog EcoPlates based on the carbon source utilization pattern, while the metabolic function of only bacteria was predicted in the present study. Nevertheless, some similar findings were noted, in that the relative abundances of amino acid metabolism, carbohydrate metabolism, and metabolism of cofactors significantly increased in RSD-treated soils. Chen et al. (2021) revealed that RSD treatments can effectively reduce the expression of antibiotic resistance genes (ARGs), including part members of multidrug, beta-lactam, macrolide, and phenicol resistance genes. We observed that most human disease functions significantly decreased during RSD treatments as compared to that in CK soils; this may be associated with the reduction of some ARGs or human pathogenic bacteria, which should be confirmed in the future by using more advanced techniques such as metagenomic sequencing and high-throughput quantitative PCR (Zhu et al., 2017). For fungi, the relative abundance of saprotrophs was also enriched in RSD-treated soils, which seems beneficial for soil quality because the increase of saprophytic fungi can promote the formation of humus and provide the required energy source for improving soil fertility (Li et al., 2021). Overall, most soil properties, such as acidification, number of pathogens, bacterial community, and functional compositions, influenced by RSD treatment were distributed more reasonably than those in the comparable healthy soil.

### RSD process combined with *Paenibacillus* sp. inoculation further enhanced the performance of plant physiological properties

In this study, we observed that RSD treatments effectively decreased the plant disease incidence and increased crop yield, which is consistent with the results of previous studies (Butler et al., 2012; Huang et al., 2016). The plant health status can also directly affect the physiological properties of fruits, such as soluble proteins and ascorbic acid, which in turn are closely related to the host health (Kruijt et al., 2005; Asensi-Fabado and Munne-Bosch, 2010). This is in line with our study finding that ascorbic acid and soluble protein contents significantly interacted with the disease incidence and population of *F. oxysporum*.

Ascorbic acid, as a multifunctional metabolic substance, not only plays a very important role in plant antioxidation and photosynthesis processes but also induces systemic resistance by affecting the biosynthesis of plant hormones (Asensi-Fabado and Munne-Bosch, 2010). For example, the low content of ascorbic acid can effectively promote plant defense response by regulating the biosynthesis of abscisic acid, jasmonic acid, and ethylene, whereas an opposite trend was found in plants with a high content of ascorbate (Kuzniak and Sklodowska, 2001; Conklin and Barth, 2004). Furthermore, soluble proteins, as an important osmotic regulator in plants, can also affect plant disease resistance by participating in various intracellular enzymatic reactions (Kruijt et al., 2005). Wang et al. (2002) and Li et al. (2015) reported that the content of soluble proteins in watermelon and potato tubers is significantly reduced after infection with *F. oxysporum* f. sp. *niveum* and *F. trichothecioides*, respectively. From the present study, for the first time, we found these interesting results that RSD-treated soils can significantly decrease the content of ascorbic acid and considerably increase the content of soluble proteins, which may play important roles in RSD-treated soil to induce plant systemic resistance.

Specifically, RSD-completed soil combined with the inoculation of beneficial species (such as *B. subtilis* SQR-N1 and *Trichoderma* spp.) has attracted increasing attention for enhancing plant disease resistance; this is mainly because RSD alone cannot always perform well to control soil-borne diseases during plant cultivation (Huang et al., 2016; Khadka and Miller, 2021; Ali et al., 2022). For example, our previous study showed that the control efficiencies of RSD on *Fusarium* wilt and *F. oxysporum* in watermelon were unsatisfactory (Liu et al., 2018). In the present study, MO_PA_-RSD-treated soil showed better performance than MO-RSD-treated soil in terms of these above-mentioned plant properties together with fruit taste (hardness and fracturability) and *F. oxysporum* disinfestation efficiency. This result indicates that the anaerobic and reductive environment, which was created during RSD treatment, combined with *Paenibacillus* sp. inoculation could further enhance the plant health performance from multiple aspects.

### Regulation of plant physiological properties by RSD combined with *Paenibacillus* sp. inoculation is closely linked with the proliferation of specific probiotic consortia

Not surprisingly, the effect of RSD on plant health performance is mainly associated with the improvement of microbial communities. Here, the dissimilarities in the relative abundances of the dominant bacterial and fungal genera were significantly and positively correlated with the differences in plant physiological properties; a higher correlation coefficient was observed between the dominant bacterial genera and plant physiological properties. Moreover, the relative abundances of ten dominant bacterial genera and three dominant fungal genera enriched in RSD-treated soils were significantly and negatively associated with the disease incidence or population of *F. oxysporum* (**Figure 5B**). These results revealed that the soil disinfestation and plant protection processes of RSD treatment were mainly mediated by the bacterial community.

Interestingly, all these dominant bacterial genera belonged to the phylum *Firmicutes*, which may play an important role in reducing soil-borne pathogens, mediating plant immunity, and inducing plant disease resistance by releasing antifungal compounds or stimulating the release of various plant hormones such as cytokinin (Lee et al., 2021; Gupta et al., 2022; Liu et al., 2022a). Herein, for example, the specific bacterial taxa (e.g, *Bacillus*, *Cohnella*, and *Paenibacillus* spp.) have been previously reported as the potential PGRPs, which have the capacity to promote plant growth by establishing defense lines against pathogen invasion in the rhizosphere (Li and Chen, 2019; Premalatha et al., 2021; Ali et al., 2022; Zhou et al., 2022). The species of *Oxobacter*, *Rummeliibacillus*, and *Thermoanaerobacterium* can suppress pathogens by releasing organic acids such as acetic acid, ethanol, and lactic acid during the fermentation of organic materials (Mowlick et al., 2013; Pang et al., 2018; Tan et al., 2019). In addition, the fungal members of *Zopfiella*, *Penicillium*, and *Talaromyces* are known as producers of antibiotics and enzymes, including zopfiellin, xylanolytic enzymes, crude ethyl acetate, and crude methanol (Soytong and Poeaim, 2015; Zhao et al., 2020; Liu et al., 2021), which may act against soil-borne pathogens and promote plant disease suppression.

Some recent studies have reported that the facilitative microbe-microbe interactions are widespread in the soil–plant system, which is primarily associated with the cross-feeding or production of secondary metabolites (such as siderophores) by microorganisms (Pacheco et al., 2019; Kramer et al., 2020). Undoubtedly, microorganisms do not individually perform suppression of plant diseases; they often look for “helpers” with facilitative interaction to build defense lines to limit the invasion of pathogens (Hu et al., 2021; Li et al., 2021). In the present study, we found a similar phenomenon wherein the relative abundances of *Paenibacillus*, *Cohnella*, *Effusibacillus*, *Rummeliibacillus*, *Oxobacter*, *Thermicanus*, and *Penicillium* in MO_PA_-RSD-treated soil were significantly higher than those in MO-RSD-treated soil. Importantly, the relative abundance of *Paenibacillus* significantly and positively interacted with the remaining microbial species. These results indicate that *Paenibacillus* sp. inoculation as a step in the RSD treatment process may be helpful to (1) promote its colonization efficiency, (2) stimulate the proliferation of other probiotic consortia, and (3) cooperate to maintain good soil and plant health. Notably, although the microbial community showed a huge effect on plant disease suppression, plants can also reshape their microbial composition by releasing a variety of root exudates (Haichar et al., 2008; Liu et al., 2018). Furthermore, our previous studies reported that the re-degradation of soil abiotic factors during plant cultivation, such as soil pH, can further induce the deterioration of microbial communities regulated by RSD (Liu et al., 2021). In the present study, the biotic and abiotic data were collected after RSD treatment; therefore, how to ensure the continuous colonization of these probiotic consortia during plant cultivation is a major task in future studies.

## Conclusions

The present study found that the efficiencies of RSD to improve diseased soil properties under a plastic shed cultivation system, such as soil acidification, pathogen abundance reduction, and bacterial community and functional compositions, were distributed more reasonably than those in the comparable healthy soil from an open-air paddy cultivation field. The anaerobic and reductive environment of the MO-RSD treatment process combined with the inoculation of facultative anaerobic functional species (*Paenibacillus* sp.) can further enhance the performance of soil health and plant physiological properties as compared to MO-RSD treatment alone. Specifically, this integrative RSD practice is not only beneficial for *Paenibacillus* sp. colonization, but it can also stimulate the proliferation of specific probiotic consortia, which ultimately and cooperatively control more soil-borne pathogens and induce plant systemic resistance. The present study contributes to the growing body of knowledge on how to promote RSD efficiency to maintain good soil health and plant disease resistance.

## Data availability statement

The datasets presented in this study can be found in online repositories. The names of the repository/repositories and accession number(s) can be found below: https://www.ncbi.nlm.nih.gov/, PRJNA890763.

## Author contributions

LLL, QS, ZCC, and XQH conceived designed research. YX, XZ, and QQD performed the experiment and collected the data. LLL and QS analysed the data. LLL wrote the manuscript. All authors contributed to the article and approved the submitted version.
