# Supplementary material for: Facilitating effects of the reductive soil disinfestation process combined with Paenibacillus sp. amendment on soil health and physiological properties of Momordica charantia
Source: Front Plant Sci. 2023 Jan 17;13:1095656. doi: 10.3389/fpls.2022.1095656 (PMC9888761; doi:10.3389/fpls.2022.1095656)
Supplement: Supplementary file 1 [file DataSheet_1.pdf]

Facilitating effects of the reductive soil disinfestation process combined with *Paenibacillus* sp. amendment on soil health and physiological properties of *Momordica charantia*

Liangliang Liu[1,2], Yi Xie[1,2], Xin Zhong[1,] Quanquan Deng[1], Qin Shao[1]*, Zucong Cai[2,3], Xinqi Huang[2,4]

[1]Engineering Technology Research Center of Jiangxi Universities and Colleges for Selenium Agriculture, College of Life Science and Environmental Resources, Yichun University, Yichun 336000, China

[2]School of Geography, Nanjing Normal University, Nanjing 210023, China

[3]Jiangsu Center for Collaborative Innovation in Geographical Information Resource Development and Application, Nanjing 210023, Nanjing Normal University, China

[4]Jiangsu Engineering Research Center for Soil Utilization & Sustainable Agriculture, Nanjing Normal University, Nanjing 210023, China

*** Correspondence:** Qin Shao

Email: shaoqin2013@126.com

Number of figures: 3

Number of tables: 2

Table S1 Primers used for real-time PCR and MiSeq sequencing

	Gene	Primers	Sequence (5'-3')	Reference
Real-time PCR	16s rDNA	Eub338 (F)	CCTACGGGAGGCAGCAG	Lane et al., (1991)
		Eub518 (R)	ATTACCGCGGCTGCTGG	Muyzer et al., (1993)
	Fungal ITS	ITS1f (F)	CTTGGTCATTTAGAGGAAGTAA	Gardes and Bruns, (1993)
		ITS2R (R)	GCTGCGTTCTTCATCGATGC	Vilgalys and Hester, (1990)
	F. oxysporum	ITS1f (F)	CTTGGTCATTTAGAGGAAGTAA	Gardes and Bruns, (1993)
		AFP308 (R)	CGAATTAACGCGAGTCCCAAC	Lievens et al., (2005)
MiSeq sequencing	16s rDNA	515F (F)	GTGCCAGCMGCCGCGG	Caporaso et al., (2011)
		907R (R)	CCGTCAATTCMTTTRAGTTT	Lane et al., (1985)
	Fungal ITS	ITS1F (F)	CTTGGTCATTTAGAGGAAGTAA	Gardes and Bruns, (1993)
		ITS2R (R)	GCTGCGTTCTTCATCGATGC	White et al., (1990)

References

Caporaso, J.G., Lauber, C.L., Walter,s W.A., Berg-Lyons, D., Lozupone, C.A., Turnbaugh, P.J., Fierer, N., Knight, R. (2011). Global patterns of 16S rRNA diversity at a depth of millions of sequences per sample. PNAS 108, 4516-4522. https://doi.org/10.1073/pnas.1000080107

Gardes, M., Bruns, T.D. (1993). ITS primers with enhanced specificity for *basidiomycetes*-application to the identification of mycorrhizae and rusts. Mol. Ecol. 2, 113-118. https://doi.org/10.1111/j.1365-294X.1993.tb00005.x

Lane, D.J., Pace, B., Olsen, G.J., Stahl, D.A., Sogin, M.L., Pace, N.R. (1985). Rapid determination of 16s ribosomal RNA sequences for phylogenetic analyses. PNAS 82, 6955-6959. https://doi.org/10.1073/pnas.82.20.6955

Lane, D.J. (1991). 16S/23S rRNA sequencing. In: Stackenbrandt, E., Goodfellow, M., (eds). Nucleic acid techniques in bacterial systematics. John Wiley and Sons, Chichester, United Kingdom Press, pp, 115-175.

Lievens, B., Brouwer, M., Vanachter, A.C.R.C., Lévesque, C.A., Cammue, B.P.A., Thomma, B.P.H.J. (2005). Quantitative assessment of phytopathogenic fungi in various substrates using a DNA macroarray. Environ. Microbiol. 7, 1698-1710. https://doi.org/10.1111/j.1462-2920.2005.00816.x

Muyzer, G., de Waal, E.C., Uitterlinden, A.G. (1993). Profiling of complex microbial populations by denaturing gradient gel electrophoresis analysis of polymerase chain reaction-amplified genes coding for 16S rRNA. Appl. Environ. Microb. 59, 695-700. https://doi.org/10.1128/aem.59.3.695-700.1993

Vilgalys, R., Hester, M. (1990). Rapid genetic identification and mapping of enzymatically amplified ribosomal DNA from several *Cryptococcus* species. J. Bacteriol. 172, 4238-4246. https://doi.org/10.1128/jb.172.8.4238-4246.1990

White, T.J., Bruns, T., Lee, S., Taylor, J.W. (1990). Amplification and direct sequencing of fungal ribosomal RNA genes for phylogenetics. In: Innis, M.A., Gelfand, D.H., Sninsky, J.J., White, T.J., (eds.). PCR protocols: a guide to methods and applications. New York, NY: Academic Press, Inc, pp, 315-322.

Table S2 Topological characteristics of microbial network in CK- and RSD- treated soils

Topological characteristics	Bacteria		Fungi	
	CK soils [a]	RSD soils	CK soils	RSD soils
Number of nodes	51	70	60	66
Number of edges	321	540	151	144
Average connectivity	12.58	15.43	5.03	4.36
Modularity	0.59	0.64	0.79	0.48
Average path length	1.18	1.23	2.82	1.19
Average clustering coefficient	0.94	0.96	0.71	0.89

[a] CK or RSD soils indicate the combinations of PS-CK and OA-CK or MO-RSD and MO_{PA}-RSD soils that are defined in Table 1.

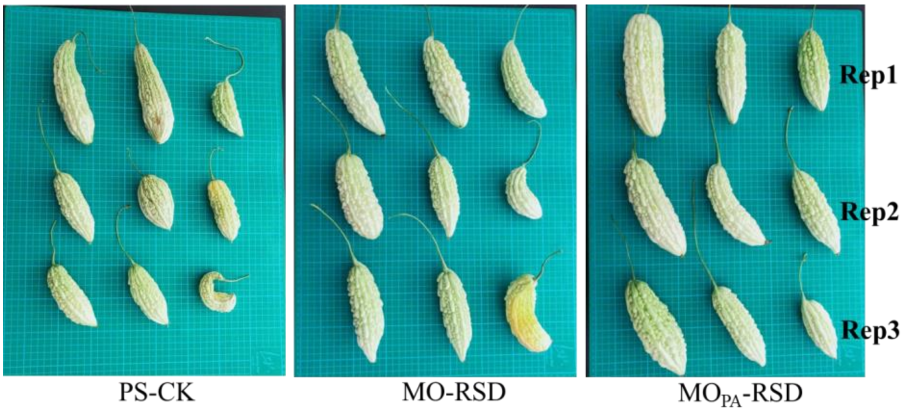

Figure S1 The weight of *momordica charantia* fruits ranked from largest to smallest in different soils. The treatment abbreviations are defined in Table 1.

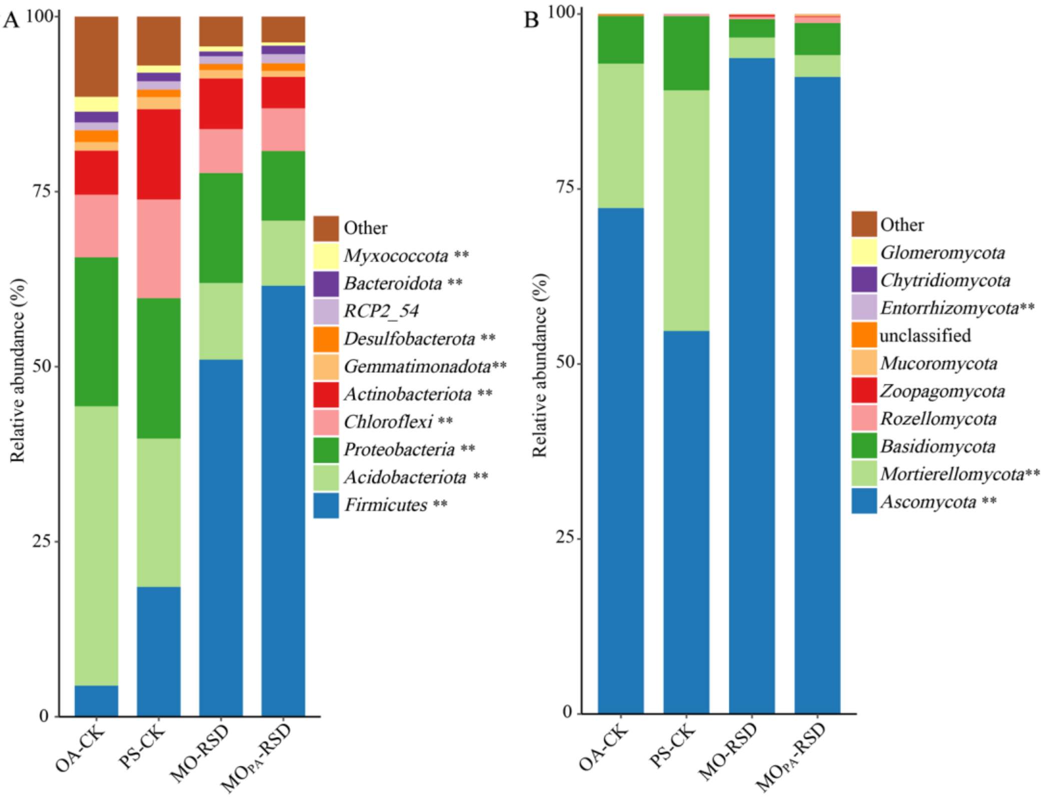

Figure S2 Composition of soil bacterial (**A**) and fungal (**B**) phyla in the different treatments. "*" ($P < 0.05$) and "**" ($P < 0.01$) indicate significant differences for a given phylum using LSD test. The treatment abbreviations are defined in Table 1.

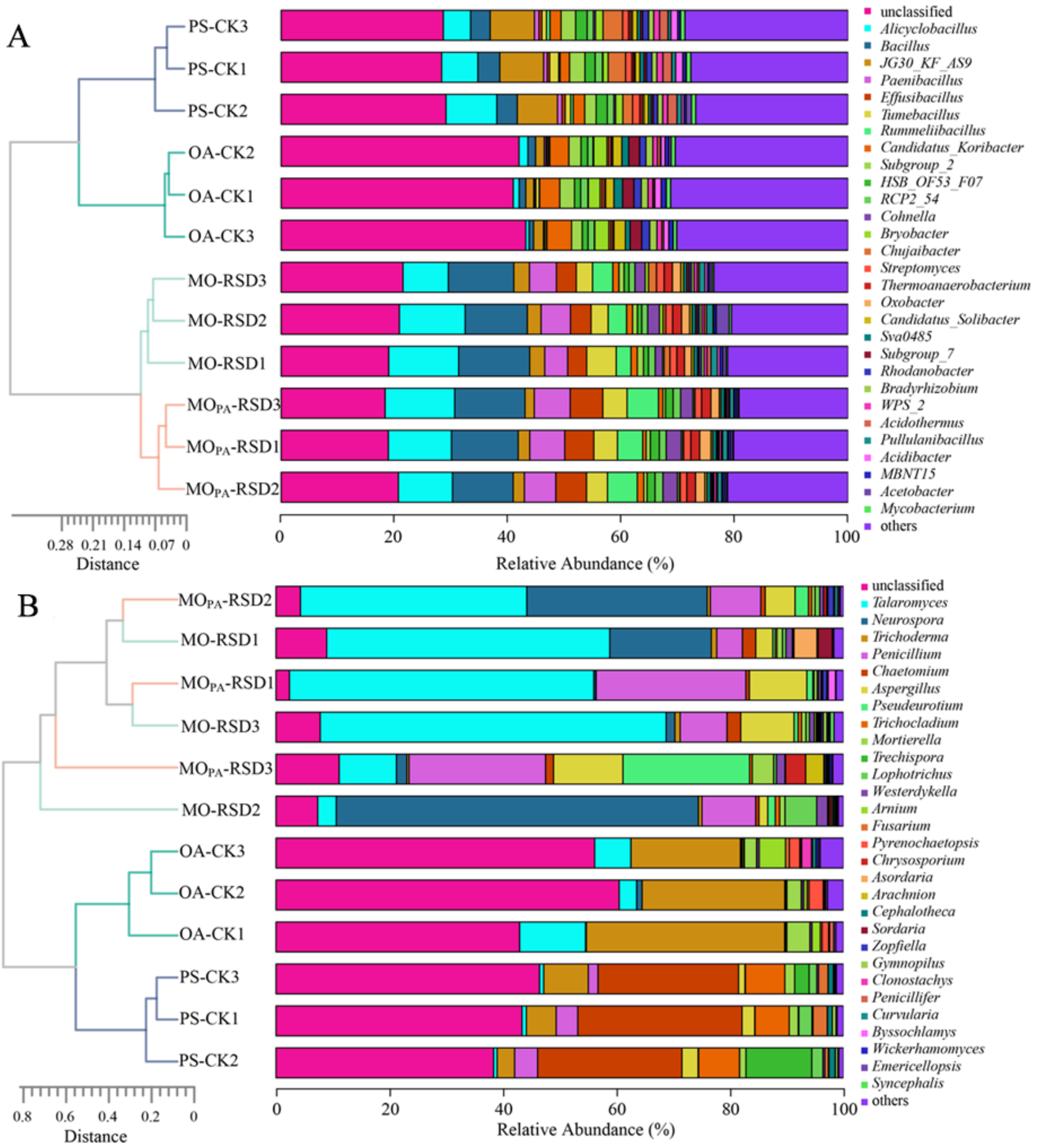

Figure S3 Composition of soil bacterial (**A**) and fungal (**B**) genera in the different treatments. Only the top 30 genera in bacteria and fungi are listed. The phylogenetic trees were determined using the neighbor-joining method. The treatment abbreviations are defined in Table 1.
